# Link-Quality Measurement and Reporting in Wireless Sensor Networks

**DOI:** 10.3390/s130303066

**Published:** 2013-03-04

**Authors:** Abdellah Chehri, Gwanggil Jeon, Byoungjo Choi

**Affiliations:** 1 School of Information Technology and Engineering, University of Ottawa, 800 King Edward Avenue Ottawa, Ontario, K1N 6N5, Canada; E-Mail: achehri@uottawa.ca; 2 Department of Embedded Systems Engineering, University of Incheon, 12-1 Songdo-dong, Yeonsu-gu, Incheon 406-772, Korea; E-Mail: bjc97r@incheon.ac.kr

**Keywords:** link-quality, measurement, wireless sensor networks, ZigBee nodes

## Abstract

Wireless Sensor networks (WSNs) are created by small hardware devices that possess the necessary functionalities to measure and exchange a variety of environmental data in their deployment setting. In this paper, we discuss the experiments in deploying a testbed as a first step towards creating a fully functional heterogeneous wireless network-based underground monitoring system. The system is mainly composed of mobile and static ZigBee nodes, which are deployed on the underground mine galleries for measuring ambient temperature. In addition, we describe the measured results of link characteristics such as received signal strength, latency and throughput for different scenarios.

## 1. Introduction

In recent years, wireless sensor networks have attracted significant attention due to their integration of wireless, computing, and sensor technology. Wireless sensor networks consist of a number of nodes that are equipped with processing, communicating and sensing capabilities. They use *ad hoc* radio protocols to forward data in a multi-hop mode of operation [1]. In the mining industry, sensor networks can provide prompt response to identification of workers entering or leaving a mine, control personnel traffic into hazardous areas to provide warning indication signals, identification of vehicles entering or leaving production areas or passing specific locations in the mine, tracking of supplies and materials, reducing the fatal accidents due to collision, monitoring of underground gases, and maintenance scheduling. The general objectives can be summarized as follows [2]:

- Real-time monitoring of gases and other parameters;
- Monitoring equipment locations and operation statuses to improve productivity and reduce fatal collision accident;
- Locating and tracking miners in case of disaster for emergency rescue operations;
- Tracking and monitoring asset equipment;
- Monitoring miner's unsafe practices and warning;

Generally speaking, the measurement of physical parameters makes the sensors the most suitable technology for monitoring and reporting important quantifiable measurements. Besides, sensors are not just limited to environment sensing. Any application involving sensing of physical parameters like sound, humidity, pressure, temperature, *etc.*, might use sensor network [3–9].

When choosing the deployment of WSN in underground mine, it may be necessary to make a compromise between conflicting requirements. The priority is to ensure a robust global network with battery-operated nodes. Therefore, these types of networks are usually developed with the following goals in mind. Firstly, the nodes must be able to communicate with other nodes via a highly reliable radio module that is compatible with the communication protocol of the network, such as the IEEE 802.15.4 standard in our case. Secondly, the network should be robust to monitor the required measurements, such as temperature, for a long time.

We deployed a wireless sensor network in experimental underground mines, CANMET (Canadian Center for Minerals and Energy Technology (CANMET) experimental mine). The network contains all elements of the architecture. To harden the test nodes and other hardware against temperature conditions, dust, and humidity present in underground mines, we designed environmental protective packaging to protect the hardware. The selected nodes by their design are fairly robust mechanically, with the battery case firmly integrated with the main processing and sensor boards [10]. Wireless communication is achieved with a transceiver compliant with the IEEE 802.15.4/ZigBee™ standard. ZigBee™ is a global standard for wireless network technology that addresses remote monitoring, environmental data measurements and control applications. ZigBee™ is an open specification that enables low power consumption, low cost and low data rate for short-range wireless connections between various electronic devices. In wireless networks, several applications and protocols utilize link quality estimations to enhance the performance of the system. However, a precise characterization of wireless links in realistic wireless networks is a challenging problem since the links experience frequent channel variations and complex interference patterns [10]. Usually, we verify the performance of any network by using simulations or experiments. In simulations, we cannot control precise packet timing, radio transmission range, memory, processing resources, and real PHY/MAC layer events [11,12]. In fact, not all simulation results are equal to the real experiments. In real experiments, we have complex environment settings and resource sharing problems.

In this paper we conducted measurements and analysis of the link quality between sensor nodes. However, the link impairments (hence quality) are intimately linked to MAC operation and therefore cannot be estimated purely on the basis of PHY measurements such as signal-to-noise ratio (SNR), even if the study integrates the MAC and PHY layers in the same problem. The MAC layer is not investigated deeply in this paper. In addition, high level measurements such as throughput and delay statistics could be better indicators of the link quality.

## 2. Wireless Sensor Network Testbed

In this section, an overview of the hardware implementation and the software protocol is given. First, a customized wireless communication test platform for evaluating wireless networking protocols is presented. A detailed description of the capabilities and limitations of the test platform is discussed [13]. The testbed consists of the following components:

- Hardware Description;
- Software Description;
- Network Architecture;
- Networks Topology;
- Node Deployment;
- WSN to Internet communication.

### 2.1. Hardware Description

The Silicon Laboratories 2.4 GHz 802.15.4 Development Board (DB) provides a hardware platform for the development of 802.15.4/ZigBee™ networks. The DB includes a Silicon Labs 8051-based MCU, a Chipcon CC2420 RF Transceiver, a JTAG (Joint Test Action Group or IEEE 1149.1 standard) connector for in-circuit programming, an assortment of programmable buttons and LEDs and a USB interface for connecting to the host computer [14].

**Figure 1.**
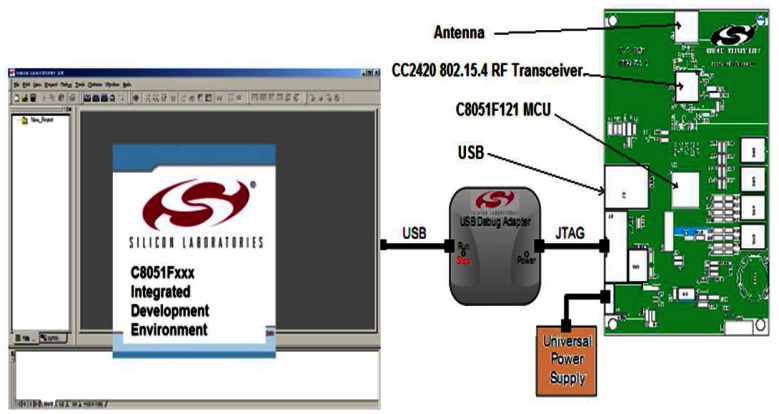
The silicon laboratories 2.4 GHz 802.15.4 mote; (**left**): development Board; (**right**): software interface.

Figure 1(a) shows a block diagram of the DB. The DB card has been developed with a minimal number of components. This is in part due to the low power-consumption requirement and in part due to the need to keep the mote size and manufacturing costs to a minimum. The core of the platform is a Silicon Labs C8051F121 (MCU) ultra-low power microcontroller. The device is quite powerful with an 8051 CPU (100 MIPS). This microcontroller can typically operate at clock frequencies up to 8 MHz with 128 kB of flash memory and 8,448 bytes of RAM.

Wireless communication is provided by the Chipcon CC2420 radio transceiver. This circuit combines low power and efficient operation with support for IEEE 802.15.4. It operates in the 2.4 GHz Industrial-Scientific-Medical (ISM) unlicensed radio frequency band, with 16 channels. It uses an automatic PCA (Parallel Channel Adapter) and address filtering. The consumption of CC240 is estimated at 19.7 mA for Rx and 17.4 mA for Tx. Automatic acknowledgment transmission is used, and a CRC criterion (Cyclic Redundancy Check) is employed to decide whether a packet was received correctly or not. The radio module is connected via an SMA connector to an omnidirectional antenna.

The DB has a total of eleven LEDs. The LEDs are used to show the state of the mote (after reset, sending a message, *etc.*) and two of them are used for power status indicators. An internal temperature sensor is included in the board with a measuring range of (−40 °*C* to +85 °*C*). The DB is powered with a 9 V battery. This work does not consider the energy consumption of the node. However this could be analyzed in the future work.

### 2.2. Software Description

The 2.4 GHz ZigBee™ development kit contains all necessary files to write, compile, download, and debug a simple IEEE 802.15.4/ZigBee™-based application. The development environment includes an IDE, evaluation C compiler, software libraries, and a several code example. The software library includes the 802.15.4 MAC and PHY layers. The ZigBee™ demonstration provides a quick and convenient graphical PC-based application. The kit also includes an adapter for programming and debugging from the IDE environment as shown in Figure 1(b). A Network Application Programming Interface (API) contains all necessary network primitives to build an 802.15.4 network from a user-defined application. A software example illustrates the MAC API. This example builds an ad-hoc 802.15.4 network using the included MAC API software library. The Silicon Laboratories 2.4 GHz development kit contains several preconfigured network topologies. These topologies are predefined and downloaded first to each node via a USB connector. For our measurements, cluster tree, star and linear topologies were separately adopted.

### 2.3. Network Architecture

**Figure 2.**
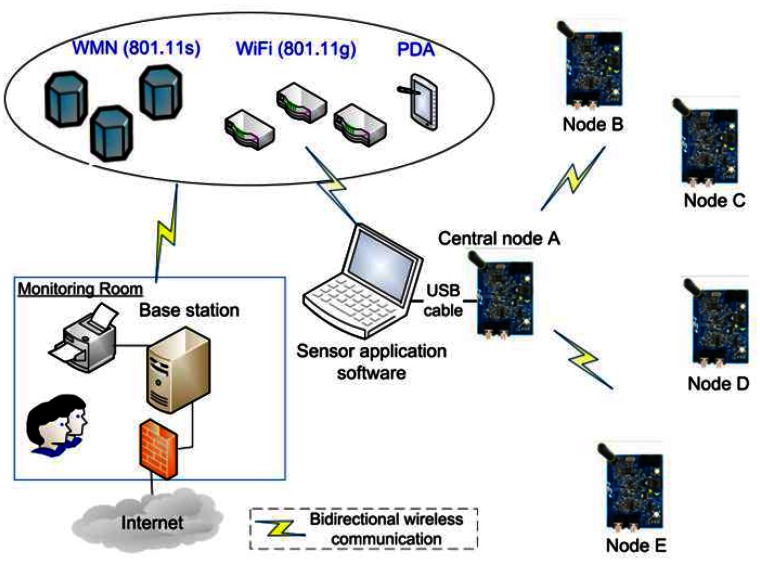
Block diagram of the heterogeneous wireless network deployment.

Wireless sensor network is used to transfer the sensor data frames from the sensor unit over a radio interface to the central node. If a radio link can be established between these modules for peer-to-peer communication, the radio modules put each sensor data frame into a radio message, send the message over the radio link, and extract the sensor data frame from the received radio message. Figure 2 shows that the sensor data are transmitted directly from the sensor node to the central node, which then transmits them to the base station. Node A is the designated master (central node) in this topology. Other nodes are Full-Function Device (FFD) routing nodes or Reduced Function Device (RFD) terminal nodes

The network organizes itself and is self-healing, *i.e.*, network nodes automatically establish and maintain connectivity among them. The static nodes (SN) are normally wall-powered and in a fixed known location, however, the mobile nodes (MN), which need to be battery-powered. The system was designed to work under normal conditions. The temperature measurement could for example prevent from fire by continually monitoring of different value.

### 2.4. Node Deployment

The deployment of sensor nodes in the physical environment may take several forms [15]. In the case of an underground mine, the deployment may be random (unexplored part of mine), at deliberately chosen spots on the top of the gallery or at a fixed position on the gallery walls. In manual deployment, the sensors are manually placed and the data are routed through predetermined path. The deployment operation may be a one-time activity, where the installation and the use of a sensor network are strictly separate activities. However, deployment may also be a continuous process, with more nodes being deployed at any time during the use of the network, for example, to replace failed nodes or to improve coverage of the network.

### 2.5. WSN to Internet Communication

The WSN has to be able to interact with other information devices, for example, a moving miner equipped with a PDA will be able to read the temperature sensors even if this node is located in different mine gallery. To this end, the WSN first of all has to be able to exchange data with such a mobile device. This scheme can be generalized to other important security parameter (carbon monoxides, or smoke concentration, for example). Therefore, for the proposed WSN monitoring system, we evaluated the performance and interoperability of sensor network with various networks such as 802.11g (WiFi) and IEEE 802.11s (wireless mesh network). In this scheme, the nodes communicate with the central node, which is connected to a laptop on site. This last one has the capability of communicating wirelessly with other computers located in a monitoring room via IEEE 802.11 networks (or wireless mesh network). The number of access points of both WiFi and wireless mesh network should be sufficient to ensure a total coverage of mine gallery.

The system is connected to the Internet through a gateway. The gateways play the role of communication between WSNs and Internet access. We use a single board computer with public IP address as a gateway in a WSN. So the ambient temperature of a mine gallery can be measured and displayed in real time no matter where we are. The global scheme of WSN mine gallery temperature monitoring is shown in Figure 2.

The system was designed to work in a normal condition. The sensors are responsible for monitoring the environment. When a fire or toxic gas is detected, the mobile sensor can utilize the information reported from sensors and find a shortest path to visit all emergency sites. Therefore this sensor-based monitoring system could provide real-time emergency-related information. In addition, compared with wire-line solution, the wireless links are able to work in accidents (fire or collapse). This huge advantage helps to save miner's life.

## 3. Measurement Setup and Results

### 3.1. Measurement Setup

**Figure 3.**
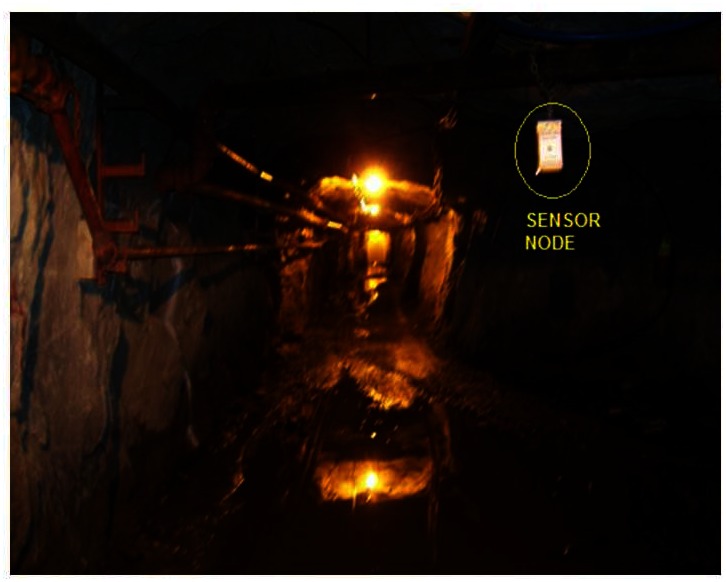
Gallery mine (CANMET).

The measurements were carried out in an underground gallery of the MMSL-CANMET laboratory mine located 540 km north of Montreal, QC, Canada. We have performed the measurements at the 70 m level. Figure 3 shows an example the node placement in the mine gallery for LOS (line-of-sight).

In this measurement configuration, the central node remained at a fixed position whereas the slave node was moved at different locations in the mine gallery. The measurements were taken for both static and moving nodes.

### 3.2. Link Characteristics

In this section, we describe some preliminary results of measured link characteristics from the testbed. Specifically, we discuss some statistics of the wireless link performance in terms of delay, received signal level, link quality indicator and throughput.

#### 3.2.1. Received Signal Strength

**Figure 4.**
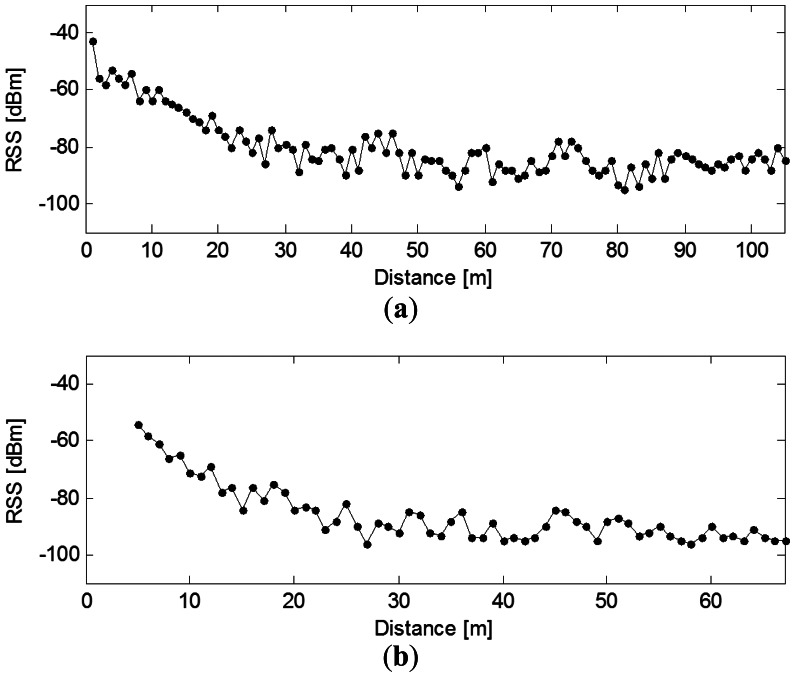
The received signal of a node *vs.* distance (**a**) LOS; (**b**) NLOS.

Figure 4 shows the received signal strength versus the distance. One can observe two regions of path loss. In the first region (1 m to 40 m), signal attenuation is about 40 dB between 1 m and 40 m, which is significant considering that the transmitter and the receiver are in line-of-sight in this case. However, the second region (from 40 m to 105 m) is characterized by small signal attenuation. This small attenuation is due to the topology of the gallery. The noise floor of these tests is located at −100 dBm and lower.

In fact, this region of the gallery is represented as a narrow corridor in which the multipath adds; therefore the signal can travel a long distance with a small attenuation. This is known as the “waveguide propagation phenomenon.”

This result agrees with other works, many of which support the use of so-called breakpoint models that employ higher values of the path loss exponent close to the transmitter. These breakpoint distances (called dp) are located in the range of 40 meters from the transmitter in the LOC scenario and in the range of 20 meters for NLOS configuration.

#### 3.2.2. Link Latency

**Figure 5.**
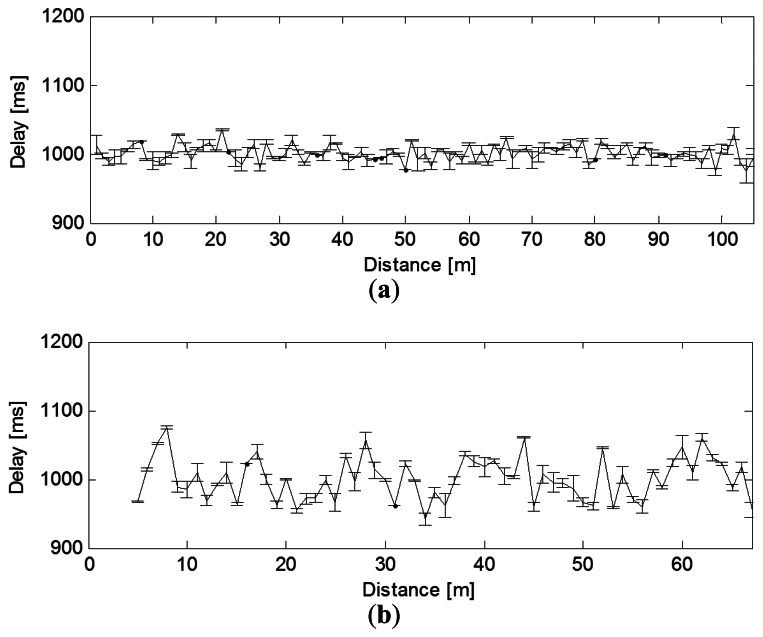
Effect of distance on delay (**a**) LOS; (**b**) NLOS.

The average end-to-end latency is the sum of transmission delay and signal sampling time, illustrated in Figure 5. Approximately, the end-to-end latency is a linear function of the payload size. The reason for this is that for a certain data packet size, the signal sampling time is much longer than the packet transmitting time.

The delay increases as the number of hops increases on the ZigBee link. In addition, the variation also increases significantly when there are more hops. However, we do not observe such a strong correlation between distance and link latency though. As shown in Figure 5, the latency does not increase from 1 m to 105 m. This delay is acceptable to most WSN monitoring applications [16].

#### 3.2.3. Throughput Analysis

**Figure 6.**
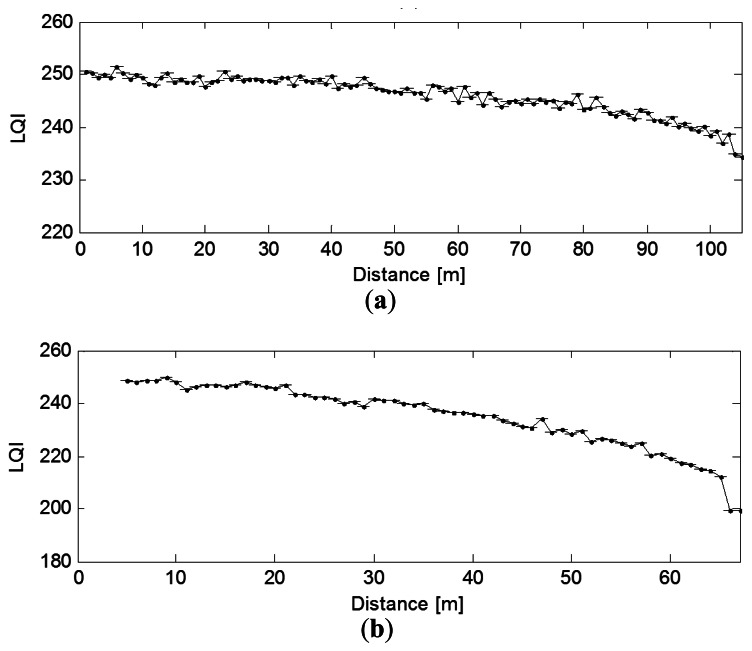
Effect of distance on LQI (**a**) LOS; (**b**) NLOS.

**Figure 7.**
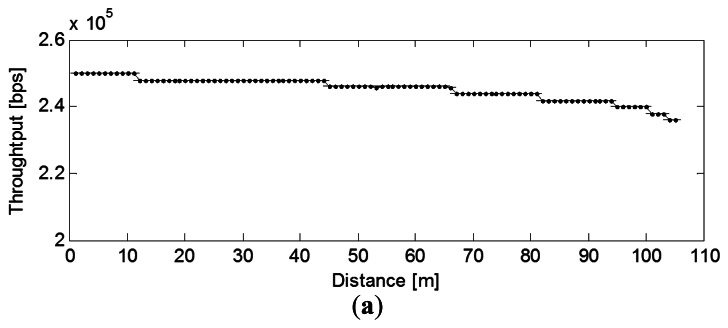

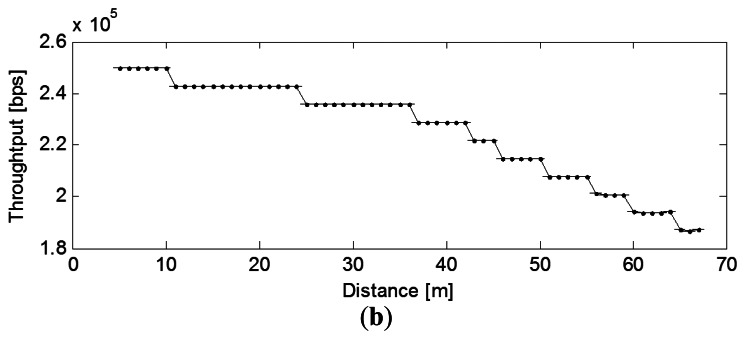
Effect of distance on Throughput (**a**) LOS; (**b**) NLOS.

The link quality indicator (LQI) is a metric introduced in IEEE 802.15.4 that measures the error in the incoming modulation of successfully received packets. We can see that the LQI degrades almost linearly with the distance for both the mote LOS and NLOS cases. The throughput, which in general mirrors the LQI pattern, also degrades with the transmitter-receiver distance, and in the LOS case it almost follows a linear decay (Figures 6 and 7).

## 4. Conclusions

Designing a wireless sensor network for data monitoring in underground mine involves several steps, including the selection of node locations and power assignments. Network performance indicators such as throughput, delay or latency and packet loss of a central node in the target area of a wireless sensor network depend on the received signal strength at the node.

These collected data in this paper could help the network designer by providing useful information. These data are used to modify node locations to ensure adequate coverage for users in the target area of service. The selected network topology and the node separation are also key parameters. The number and distribution of such test points depend upon the size of the underground mine gallery area as well as its physical topology and anticipated number of miners. Proper selection of preliminary sensor node locations is also important for an effective site survey and design.

In this paper, an experimental deployment of a WSN in the underground mine gallery has been described. The performances of ZigBee sensor networks over real measurement configurations have been presented. Firstly, we evaluate the interoperability of wireless sensor network with various networks such as IEEE 802.11g (WiFi), IEEE 802.11s (wireless mesh network) and Internet. In addition, we describe the measured link characteristics from the testbed. Specifically, we discuss some statistics of the wireless link performance in terms of delay, received signal loss, link quality indicator and throughput.
